# Aggressive Vaccine-Induced Immune Thrombocytopenia and Thrombosis in a Young Woman with a Past Mild SARS-CoV-2 Infection

**DOI:** 10.3390/reports7010017

**Published:** 2024-02-23

**Authors:** Filippo Luciani, Maria Cristina Caroleo, Alfredo Zanolini, Lucio Taranto, Pino Pasqua, Alfredo Petrone, Manuela Colosimo, Roberto Cannataro, Erika Cione

**Affiliations:** 1 Infectious Diseases Unit, Annunziata Hospital, 87100 Cosenza, Italy; filippoluciani@gmail.com; 2 Department of Health Science, University of Magna Graecia at Catanzaro, 88100 Catanzaro, Italy; 3 Radiology Unit, Annunziata Hospital, 87100 Cosenza, Italy; alfredozanolini@gmail.com (A.Z.); luciotaranto@gmail.com (L.T.); 4 Intensive Care Unit, Annunziata Hospital, 87100 Cosenza, Italy; p.pasqua@virgilio.it; 5 Neurology Unit, Annunziata Hospital, 87100 Cosenza, Italy; alpetrone@gmail.com; 6 Department of Microbiology and Virology, Pugliese Ciaccio Hospital, 88100 Catanzaro, Italy; manuelacolosimo@hotmail.it; 7 GalaScreen SRL, University of Calabria, 87036 Rende, Italy; r.cannataro@gmail.com (R.C.); erika.cione@unical.it (E.C.); 8 Department of Pharmacy and Health and Nutritional Sciences, University of Calabria, 87036 Rende, Italy

**Keywords:** VITT, ChAdOx1 nCov-19 vaccine, stroke, transtentorial uncal herniation

## Abstract

Vaccine- induced immune thrombocytopenia and thrombosis (VITT) is a rare adverse event occurring after immunization with adenoviral vector-based vaccines against SARS-CoV-2. This life-threatening condition is characterized by thrombocytopenia, systemic activation of coagulation, and anti-platelet factor 4 antibodies, often resulting in extensive venous thrombosis. Arterial thrombosis is less common and mainly affects the aorta, peripheral arteries, heart, and brain. Several cases of ischemic stroke have been reported in VITT patients, frequently being associated with large vessel occlusion (LVO). Here, we present a case of aggressive VITT in a 46-year-old woman with a past mild SARS-CoV-2 infection, who was admitted with a left-middle cerebral artery (MCA) territory stroke and thrombocytopenia eight days after her first dose of the ChAdOx1 nCoV-19 vaccine. The patient developed a diffuse arterial thrombosis with concomitant thrombotic events in the intrahepatic portal branches. The patient’s clinical condition worsened rapidly due to a significant enlargement of the ischemic cerebral lesion in the left hemisphere, cerebral herniation, and incipient hydrocephalus requiring decompressive neurosurgery with an unfavorable outcome. Our observations may be indicative of a stroke variant in VITT and highlight the diverse clinical manifestations of the syndrome.

## 1. Introduction

Vaccine-induced immune thrombocytopenia and thrombosis, or VITT, is recognized as a rare adverse event mainly occurring after immunization with adenoviral vector-based vaccines against SARS-CoV-2 [1]. This suggests a common mechanism that is unique to these products; indeed, reports of this syndrome following inoculation with an mRNA COVID-19 vaccine have been even more rare [2]. ChAdOx1 nCoV-19 and Ad26.COV2. S vaccines contribute to 90 and 8.5% of cases, respectively [1]. The incidence of VITT ranges from 1 in 26,500 to 263,000 people after the administration of an adenoviral vector vaccine, with an overall incidence of about 1 in 100,000 [3]. From a clinical viewpoint the concomitant occurrence of low platelet counts and thrombosis is an unusual event which has been previously restricted to a specific pathologic condition such as disseminated intravascular coagulation (DIC), antiphospholipid antibody syndrome, vasculitis, malignancies, heparin-induced thrombocytopenia and thrombosis (HITT), paroxysmal nocturnal hemoglobinuria myeloproliferative disorders, and pre-eclampsia. These thrombotic thrombocytopenic disorders share common pathophysiologic mechanisms, suggesting that vaccines containing an adenovirus vector backbone might induce thrombocytopenia and thrombosis through a process involving either the immune system or the vasculature [4]. Notably, patients with VITT develop antibodies against the platelet factor 4 (PF4), thus mirroring HITT [5,6]. However, the presence in this population of both high D-Dimer and low fibrinogen levels could also draw an analogy with DIC. The pathobiology of VITT is still incompletely understood. One proposed model suggests that VITT triggers a similar autoimmune disorder to HITT and leads to DIC. In this model a component of the vaccine might interact directly with PF4 leading to a novel autoimmune humoral response which, in turn, induces platelet activation and an hypercoagulative state [7,8]. Regarding vaccination with ChAdOx1 nCoV-19, VITT can occur in response to the first or second dose, with most cases originating from the first ones [3]. Host risk factors have also been postulated including younger age, female sex, and comorbidities [9]. However, the true risk factors for the condition remain unclear. Hallmarks of VITT are the time window for the onset of symptoms which ranges from 5 to 42 days’ post vaccination, thrombocytopenia, very high D-dimer levels, often with concomitant low fibrinogen, and thrombotic events at uncommon sites [6]. Standard criterion for confirming diagnosis in patients with suspected VITT is positivity for serum antibodies against platelet factor 4 (PF4) on ELISA. However, in multiple cases negative or ambiguous results have been obtained [5,10]. Several biases including different ELISA assays, lack of standardization in the functional platelet tests or sample timing might account for these results. Both diagnosis and guide treatments are supported by imaging approaches [11,12]. Severe cerebral venous sinus thrombosis (CVST) accounts for about 50% of clinical presentations, bringing with it cerebral oedema, an increase in intracranial pressure, and secondary intracranial hemorrhage with high mortality if severe thrombocytopenia concomitantly occurs. Thrombotic events can also affect alternative anatomical sites including portal and splanchnic veins, deep veins, and lungs. Arterial thrombosis appears to be less frequent and mainly affects the aorta, peripheral arteries, heart, and brain [13]. Herein, we present a case of aggressive VITT in a 46-year-old woman with a past mild SARS-CoV-2 infection, who was admitted with a left-middle cerebral artery (MCA) territory stroke and thrombocytopenia eight days after her first dose of ChAdOx1 nCoV-19 vaccine. She developed arterial thrombosis involving the left internal carotid, the ipsilateral sylvian axis, and the anterior cerebral artery. Concomitant thrombotic events in the intrahepatic portal branches also occurred. The patient’s clinical condition worsened rapidly due to a significant enlargement of the ischemic cerebral lesion in the left hemisphere, cerebral herniation, and incipient hydrocephalus requiring decompressive neurosurgery. The patient showed an unfavorable outcome because of respiratory arrest 48 h after surgery.

## 2. Detailed Case Description

**Figure 1:**
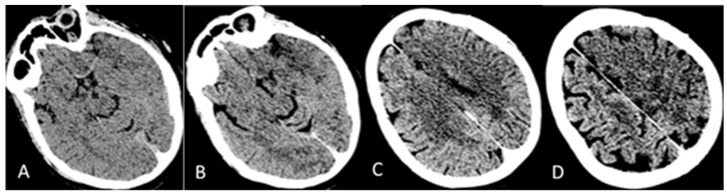
Brain CT on day one. (**A**) Vessel hyperdensity on the left of thrombotic significance and bifurcation of the siphon, the M1, and the A1–A2 tracts; (**B**–**D**): Cortico–subcortical temporo-insular and fronto-cingulum-parietal hypodensity. ASPECT score 4.

A 46-year-old Caucasian woman was admitted to the Emergency Medicine Unit at the local hospital with a complaint of sudden weakness in the right side of the body and speech difficulty on 4 June 2021. The patient was in good general clinical condition and with no significant pathologies of both congenital and acquired origins in her past medical history or upon physical examination. Her family history was also negative for the occurrence of thrombotic events. In December 2020, she developed a SARS-CoV-2 infection in a mild form. On 27 May 2021, she received an anti-COVID-19 vaccination with the first dose of ChAdOx1 nCoV-19. Following a head Computed Tomography (CT) and CT angiography of the cerebral vessels and the intracranial and epiaortic tracts, a thrombotic occlusion of the left-middle cerebral artery was revealed. Laboratory blood tests, performed in the local hospital, showed the presence of thrombocytopenia. For this reason, the patient was not eligible for intravenous fibrinolysis, and she was therefore transferred, on 5 June 2021, to the Annunziata Hospital, Cosenza, which is equipped with a Second Level Stroke Unit, for possible mechanical thrombectomy. Upon arrival in the emergency room, the patient appeared alert, with global aphasia, central-type right seventh cranial nerve deficit, palsy of the right upper limb, and severe paresis of the right lower limb, with an NIHSS score equal to 18. Her vital signs were in the normal range (BP 110/79 mmHg; HR 68 bpm R; axillary temperature 36.3 °C; O_2_ saturation 98% at room temperature). Blood analysis revealed the presence of thrombocytopenia, an increase in the D-dimer compared to reference values, and fibrinogen and glycemia in the normal value range (Table 1). A brain CT showed an extensive hypodense area suggestive of acute ischemic lesion with a frontal–cingulate cortical–subcortical site, temporo-insular, and marginally left parietal. Initial disappearance of the cortico–subcortical interface (ASPECT score: 4), smoothing of the left fronto-temporo-insulo-parietal cortical sulci, hyperdensity of the supraclinoid tract of the left siphon and the A1 and M1 ipsilateral tracts, of probable thrombotic significance, were evident, as is shown in Figure 1A–D.

**Figure 2:**
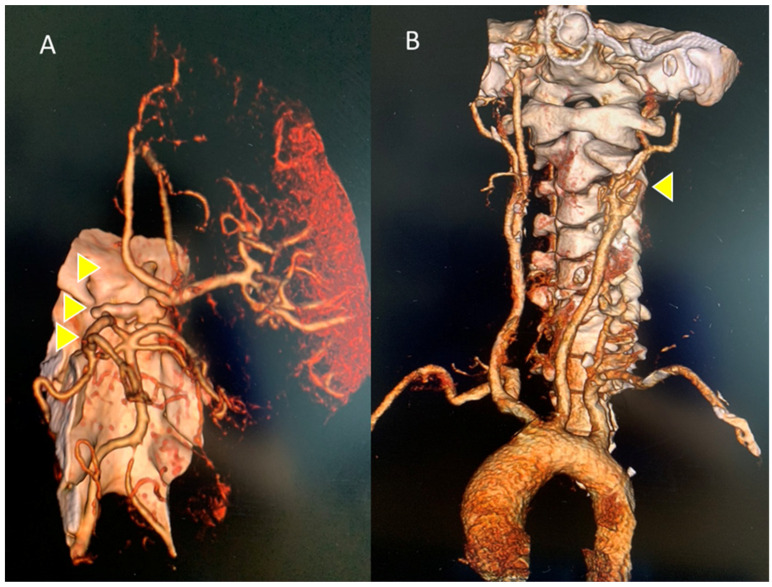
Intracranial and epiaortic angio-CT: (**A**) left anterior circulation occlusion: intracranial internal carotid, middle cerebral (MCA), anterior cerebral arteries; (**B**) lack of opacification on the left of the internal carotid artery a few mm from the origin. Thrombotic occlusion areas are indicated by yellow arrows.

CT angiography of the epiaortic and intracranial arterial vessels showed a lack of opacification of the left internal carotid starting from a few millimeters after its origin, and of the ipsilateral sylvian axis and the anterior cerebral artery, with a poor collateral circulation of compensation. The perfusion angio-CT documented hypoperfusion, with a delay in the TTP at the fronto-insulo-parietal and left nucleocapsular site, in which an almost equivalent area of reduced MTT and CBV was inscribed, as per the ischemic core, and poor MTT/CBV mismatch, are is shown in Figure 2A and Figure 2B, respectively.

**Figure 3:**
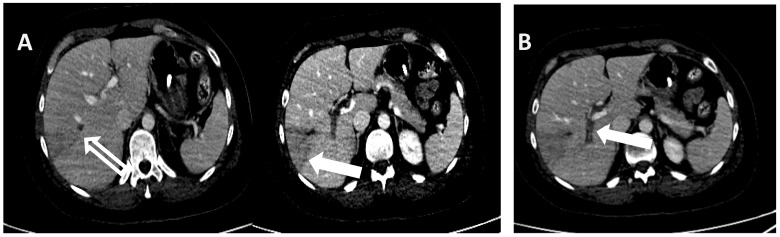
Thrombosis of the right segmental branch of the portal vein is associated with partial thrombosis of the related branch of the ipsilateral hepatic vein (empty arrow) panel (**A**). An area of parenchymal hypoperfusion involving V and VIII segments is seen (full arrow) panel (**B**).

The patient then underwent blood count monitoring. As shown in Table 1, the results revealed the persistence of thrombocytopenia and the presence of normocytic anemia with haptoglobin and bilirubin in the normal range as shown by laboratory data (Table 1). A chest and abdomen CT was therefore performed. The imaging test showed sectorial thrombosis of the right intrahepatic portal branches (VI and VII segment) and a lack of opacification in the left segmental ones, with an associated alteration in perfusion in both the right and left lobe, and some areas constantly hypodense at the level of the aforesaid segments, with a subglissonian location and a predominantly triangular morphology, probably of ischemic significance. Thrombosis of the hepatic veins or the inferior caval vein is not evident (Figure 3).

Anamnestic, clinical, radiological, and laboratory data suggested a diagnosis of VITT. The anti-PF4 ELISA test was positive (Table 1), thus confirming the suspicion of VITT.

Differential diagnosis with other commoner causes of thrombotic events such as cardiac structural abnormalities, paroxysmal nocturnal hemoglobinuria (PNH), and antiphospholipid syndrome was also posed. Based on the patient’s clinical history and on the physical examination, cardiac structural abnormalities and PNH were ruled out, while IgG and IgM cardiolipin antibody titers and IgG and an IgM β_2_-glycoprotein-I antibody titer in the negative range of values (Table 1) confirmed the absence of antiphospholipid syndrome. Laboratory results also revealed an increase in both anti-thyroglobulin and thyroid peroxidase antibody titers with concomitant level of TSH-receptor antibodies in the normal range underscoring a condition of hypothyroidism.

**Figure 4:**
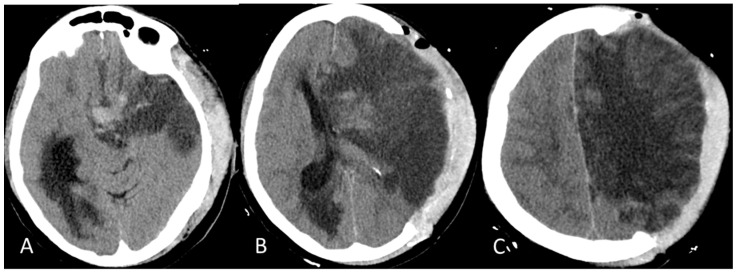
CT Brain on last day. (**A**) Transtentorial uncal herniation compressing the midbrain bridge from left to right; blood secretion in the nucleocapsular and subnucleocapsular regions; (**B**) significant compression on the III and on the left SV, with significant shift to the right and hydrocephalus of the right SV with signs of transependymal CSF transudation; and (**C**) clear and diffuse cortico-subcortical hypodensity of ischemic-edematous significance in the anterior cerebral artery (ACA) and middle cerebral artery (MCA) vascularization territories, from “malignant” cerebral infarction; the artery territories partly preserved posterior cerebral (posterior occipitotemporal) results of left fronto-parietal decompressive craniotomy with brain herniation (“brain fungus”).

The patient was then admitted to the Neurology Unit. She appeared alert, with severe aphasia and right hemiplegia. She did not show signs of hemorrhagic diathesis, no petechiae, nor bleeding from the sampling sites. Therapy with 40 mg intra-venous (i.v.) dexamethasone; i.v. High Dose IgG (IgG-HD) 1 g/Kg once daily for two days; and subcutaneous (s.c.) fondaparinux 2.5 mg once daily was started. The patient’s clinical condition deteriorated rapidly. She went into a GCS 5 coma on the second day, with pupillary anisocoria from left > right, right hemiplegia, tachypnea, and severe respiratory failure (at EGA: pO_2_ 64; pCO_2_ 19; pH 7.5). A brain CT scan was then performed revealing a more significant enlargement of the ischemic cerebral lesion in the left hemisphere with transtentorial uncal herniation to the right of the third ventricle and the left lateral ventricle, obliteration of the suprachiasmatic cistern, and enlargement of the right temporal horn, as from incipient hydrocephalus, as is shown in Figure 4A–C.

She was transferred to the intensive unit; in which she underwent neurosurgery for cerebral decompression with an unfavorable outcome because of respiratory arrest 48 h after surgery.

## 3. Discussion

This case report documented a rapid and fatal thrombotic ischemic stroke with transtentorial uncal herniation in a young woman with a past mild SARS-CoV-2 infection who received a first dose of the ChAdOx1 nCoV-19 vaccine. Laboratory tests revealed the presence of thrombocytopenia, increased serum levels of D-dimer, and positivity for platelet factor 4. As per the American Society of Hematology guidelines for VITT diagnostic criteria, we ascribed the thrombosis to vaccine administration due to the symptoms-onset time window, the past medical history of our patient showed no significant inherited or acquired coagulopathies, the occurrence of both thrombocytopenia (platelet count < 150 × 10^9^/L), the markedly elevated D-dimer (more than four times the upper limit of normal), and the presence of serum antibodies against platelet factor 4 on ELISA assay. ChAdOx1 nCoV-19-associated VITT generally occurs in the venous system, while arterial thrombosis seems to be a rare event. In that regard, large-vessel occlusion (LVO) has been reported in patients following ChAdOx1 nCoV-19 vaccination occurring mainly in the MCA-M1 segment followed by intracranial ICA or the posterior circulation stroke subtypes [14,15,16,17]. Our patient presented diffuse brain arterial thrombotic events encompassing the left internal carotid, the ipsilateral sylvian axis, and the anterior cerebral artery as revealed by CT angiography. She also showed a dramatic increase in D-dimer levels suggesting the presence of thrombotic manifestations even in extracerebral areas. Chest and abdomen CT scans documented the sectorial thrombosis of right intrahepatic portal branches and the lack of opacification in the left segmental ones, thus confirming the involvement of additional vascular compartments. In our patient, both clinical manifestations and imaging analysis suggested an unusual severity of VITT syndrome. Currently, the pathogenesis of VITT is thought to be likely related to the formation of antibodies against platelet antigens that, in turn, lead to thrombocytopenia and thrombosis [1]. Nevertheless, other factors contributing to the syndrome’s pathogenetic mechanism cannot be ruled out [4]. Anti-PF4 antibodies can also be detected in patients with mild and severe COVID-19 infection, and they are correlated with infection severity [18]. However, the impact of a prior SARS-CoV-2 infection on endothelial homeostasis, even in the setting of patients with mild infection, as in our case, is still not fully understood. The injury to the endothelial cells mediated by the viral infection was established quite early as the putative mechanism underlying the so-called post-COVID syndrome [19,20], but the presence of chronic endothelial impairment leading to an increased risk of cardiovascular and thrombotic complications in COVID-19 convalescents it is still an open question. In this framework, a recent study [21] showed the occurrence a long-term rise in soluble-form E-selectin in healthy subjects with past mild-to-moderate SARS-CoV2 infection, raising the hypothesis of an underlying susceptibility to thrombogenic events. In this patient, blood count monitoring also revealed the presence of normocytic normochromic anemia. A possible reason for this condition is the concomitant hypothyroidism indicated by the increases in both the anti-thyroglobulin and thyroid peroxidase antibodies titers with the TSH-receptor antibodies level being in the normal range. In hypothyroidism, normocytic normochromic anemia is a frequent clinical condition since thyroid hormones stimulate the proliferation of erythrocyte precursors both directly and via the enhancement of erythropoietin production. A further possible reason for the occurrence of normocytic normochromic anemia in this patient could be conditions such as hypermenorrhea in a cycle of normal duration or menorrhagia. We ruled out a process of hemolysis since both the bilirubin and haptoglobin levels were in the normal range. Treatment of a stroke in the setting of VITT is still challenging. The mainstay of treating VITT patients is therapeutic anticoagulation, which should not be postponed as they can deteriorate rapidly. Safe anticoagulants include oral and parenteral direct thrombin inhibitors (DTI), oral factor Xa inhibitors, and fondaparinux [4]. Considering the similarities between VITT and HITT, the use of heparin is not recommended for initial treatment, although this drug does not appear to be dangerous in the vast majority of VITT patients [22]. The modulation of autoimmune response through the administration of i.v. IgG-HD is the second pillar of VITT treatment. This therapeutic strategy improves thrombocytopenia in patients developing VITT and various thrombotic complications after inoculation with the ChAdOx1 nCoV-19 vaccine as reported in various case studies [23,24]. Pertaining to our case, the presence of an already evident extensive ischemic lesion on the brain CT and the absence of mismatch and, therefore, of salvable tissue on perfusion CT advised against carrying out systemic fibrinolysis with alteplase, which is not recommended in the case of an ASPECT score lower than seven because of the high bleeding risk. Hence, a therapy with dexamethasone i.v. 40 mg once daily; IV Ig-HD 1 g/Kg once daily for two days; and SC fondaparinux 2.5 mg once daily was performed. Despite the initiation of therapeutic regimen, the patient’s clinical condition worsened rapidly. She went into a GCS 5 coma and developed cerebral herniation and obstructive hydrocephalus. Both clinical manifestations and imaging analysis met the indication criteria for decompressive craniectomy, therefore the patient underwent this neurosurgical procedure with an unfavorable outcome due to respiratory arrest.

## 4. Conclusions

In conclusion, we reported a severe and fatal case of VITT in a young adult woman who received a first dose of the ChAdOx1 nCoV-19 vaccine. Several issues deserve consideration such as the clinical history of past mild SARS-CoV-2 infection which could predispose the patient to the occurrence of severe thrombotic events after administration of adenoviral-based vaccine; the diffuse brain artery thrombosis encompassing the left internal carotid, the ipsilateral sylvian axis, and the anterior cerebral artery with concomitant sectorial intrahepatic portal branches thrombosis; the development of transtentorial uncal herniation; and the fast unfavorable clinical evolution. A further aspect that should be taken into consideration is the unresponsiveness to therapeutic regimen. In that regard, plasma exchange has been proposed as a possible rescue therapy in severe VITT; however, this procedure needs further validation [25,26]. Collectively, these findings might be indicative of a VITT stroke variant and highlight the various clinical manifestations of this syndrome related to immunization with adenoviral vector-based vaccines.

## Figures and Tables

**Table 1:** Time course of blood count and biochemical parameters.

		June 2021				
Blood Count and Biochemical Parameters	5th	9th	13th	16th	17th	Reference Value
Erythrocytes (10^9^/mL)	3.25	3.47	2.96	2.97	2.61	4–5.2
Hemoglobin (g/dL)	9.7	10.5	8.6	9.1	8	12–16
Hematocrit (%)	27.5	27.8	24.7	24.4	21.8	36–46
Mean corpuscular volume (fl)	84.6	80.1	83.4	82.2	83.5	80–100
Mean corpuscular hemoglobin (pg/mL)	29.8	30.3	29.1	30.6	30.7	27–32
Mean corpuscular hemoglobin concentration (%)	35.3	37.8	34.8	37.3	36.7	32–36
Platelets (10^6^/mL)	25	23	14	37	27	150–450
Platelets volume mean (fl)	-	-	10.7	10.5	11.4	8–13
Breadth of the erythrocyte distribution (%)	14.2	13.6	14.4	13.9	14.6	2–15
WBC (10^6^/mL)	17.7	8.9	11.6	9.6	12.5	4–11
Neutrophils (%)	85.1	93.9	84.5	79.6	87.5	40–80
Lymphocytes (%)	12.8	4.6	12.5	16.8	10.7	20–40
Monocytes (%)	1.7	1.5	2.8	2.5	1.4	2–8
Eosinophils (%)	0.3	0	0	0.6	0.2	1–4
Basophils (%)	0.1	0	0.2	0.5	0.2	0–1
Neutrophils (10^6^/mL)	15	8.3	9.8	7.6	10.9	2–8
Lymphocytes (10^6^/mL)	2.2	0.4	1.4	1.6	1.3	1.5–3.5
Monocytes (10^6^/mL)	0.3	0.1	0.3	0.2	0.1	0.4–0.8
Eosinophils (10^6^/mL)	0	0	0	0	0	0.1–0.4
Basophils (10^6^/mL)	0	0	0	0	0	0.02–0.05
Glycemia (mg/dL)	90					76–106
D-dimer (mg/L)	70.75				8.25	0–0.5 FEU
Fibrinogen (mg/dL)	245			433	423	150–450
Anti-PF4 (ELISA) **^#^**				1.95 _OD_		
Haptoglobin (mg/dL)				35		30–200
Total Bilirubin (mg/dL)				1.05		0.4–1.2
Anti Thyroid peroxidase antibodies (UI/mL)		51.43				<35
Anti Tyreoglobulin antibodies (U/mL)		192.2				<116
Anti TSH receptor antibodies (UI/L)		0.27				0.27–1.8
Anti b_2_ glicoprotein antibodies (IgM; U/mL)		0.5				<7
Anti b_2_ glicoprotein antibodies (IgG; U/mL)		2.2				<7
Anti cardiolipin antibodies (IgG; GPL-U/mL)		1.2				<10
Anti cardiolipin antibodies (IgM; MPL-U/mL)		0.7				<10

WBC = white blood cells; **^#^** positive threshold: <0.40 OD for the Lifecodes PF4 IgG assay.

## Data Availability

The data presented in this study are available on request from the corresponding author.
